# Assessment of priorities, quality, and inclusivity of digital therapeutics trials in China

**DOI:** 10.1038/s41746-025-01477-6

**Published:** 2025-02-04

**Authors:** Ziming Wang, Xinxin Xia, Weijia Lu, Yuguo Ye, Jin Xu

**Affiliations:** 1 China Center for Health Development Studies, Peking University, Xueyuan Road, Beijing, 38 China; 2 Hainan Institute of Health Development Studies, Xueyuan Road, 3 Haikou, China

**Keywords:** Databases, Clinical trial design, Technology, Health services

## Abstract

Digital therapeutics (DTx) are software-driven solutions for prevention, treatment, and management of medical conditions. Despite a pro-DTx momentum in China, global DTx trial assessments overlooked the country. We identified 756 DTx trials in China and analyzed their characteristics and quality parameters. Over 70% were funded by governments, hospitals, and universities, with tertiary hospitals in eastern China leading most trials. 44.8% used automated DTx, with 39.2% DTx-guided. Most trials focused on management (52.5%) and treatment (38.1%), with few on prevention (9.4%). Mental, behavioral, or neurodevelopmental disorders represented the leading condition category of focus. Recent declines in median sample size, median duration, and mean number of sites were noted. Only 18% of trials were at low overall risk of bias. While recognizing the rapid development of DTx trials in China, we call for better trial design and methodological rigor, prioritization of preventive and primary care, wider condition category scope, and higher inclusivity.

## Introduction

Digital therapeutics (DTx) are software-driven evidence-based health interventions with positive therapeutic impact on certain medical conditions^1,2^. By facilitating evidence-based practices through information technologies that can be fully automated for patient self-care or under the support of healthcare professionals^3^, DTx can significantly improve access to quality health services^4^. DTx also offer novel approaches for medical conditions where traditional healthcare services and medications are unavailable or ineffective^5^, and can be much more cost-effective than conventional treatment^6,7^.

China has been actively promoting digital health technology and services, embedded in grand national initiatives such as “Healthy China”, “Digital China”, and national five-year plans^8^. Local governments have also introduced measures to foster a research and regulatory environment conducive to the development and application of DTx^9–11^. For these efforts to be effective, similar to other national regulations, DTx require government authorization (either clearance or approval, depending on the risk posed to patients) before commercialization^12^, which needs to be informed by high-quality clinical trials^13^.

Public healthcare institutions are the dominant providers of health services in China. However, their revenue primarily comes from service charges reimbursed partially by social health insurance, which includes mandatory health insurance for formal employees and voluntary health insurance heavily subsidized by general government revenue for residents (i.e. those outside formal employment)^14^. Provider payment is predominantly fee-for-service for ambulatory care and case-based for inpatient care. Demonstrated effectiveness of DTx in disease prevention and management at home may reduce the need for extensive hospital stays. Published Chinese expert consensuses on DTx have consistently highlighted a lack of standardization in product and trial design^15–18^.

Recent studies systematically reviewed the characteristics and quality of DTx trials registered in US- and Europe-based registries and agency websites, each using different selection criteria (Supplementary Table 1). Kumar et al. analyzed 68 published clinical studies of 20 Food and Drug Administration (FDA)-authorized prescription DTx available in the US, using highly focused search terms (i.e. brand names, and FDA product codes) on Clinicaltrial.gov, PubMed, the Digital Therapeutic Alliance, and manufactures’ websites^19^. They assessed trial quality parameters and patient demographics and concluded that prescription DTx trials often lack rigor and inclusivity. Also using focused search terms (i.e. “DTx”, “digital therapeutic”, and “digital intervention”), Yao et al. found 280 DTx trials and identified three types of DTx, namely, disease treatment (50.4%), disease management (42.9%) and health function improvement (6.8%). The study segmented DTx trial development into the embryonic stage (2002–2016), the exploration stage (2017–2020), and the rapid development stage (2020 onwards)^20^. It also highlighted a lack of a standardized system for clinical design.

Miao et al. used a broader range of search terms and identified 449 DTx trials on FDA-regulated device products^21^. This study highlighted issues with inclusivity (e.g. those with comorbidities, pregnant women, children, or those not fluent in English) that affect their real-world applicability. It reported a primary focus on nervous system diseases and nutritional and metabolic diseases. Masanneck and Stern further included premarket trials and identified 5889 DTx-related trials. They noted a concentration on mental health DTx, observed an increase in the mean sample size, and emphasized the need for more robust trial designs^22^.

Recent studies on DTx trials overlooked the Chinese Clinical Trial Registry (ChiCTR), where most clinical trials in China are registered. Considering the pro-DTx momentum in China, such omission means neglection of both a potentially powerful source of DTx innovation and of how well DTx trials address the health needs of the Chinese 1.4 billion population. To fill such important knowledge gaps, we conducted an overview of the DTx trial landscape in China, by identifying and analyzing DTx trials registered on ChiCTR or ClinicalTrials.gov. Building on previous literature about DTx trials globally and considering the characteristics of DTx in China, we examined the trials in terms of key features of interventions, inclusivity, and quality of the Chinese DTx trials.

## Results

### General characteristics

**Fig. 1:**
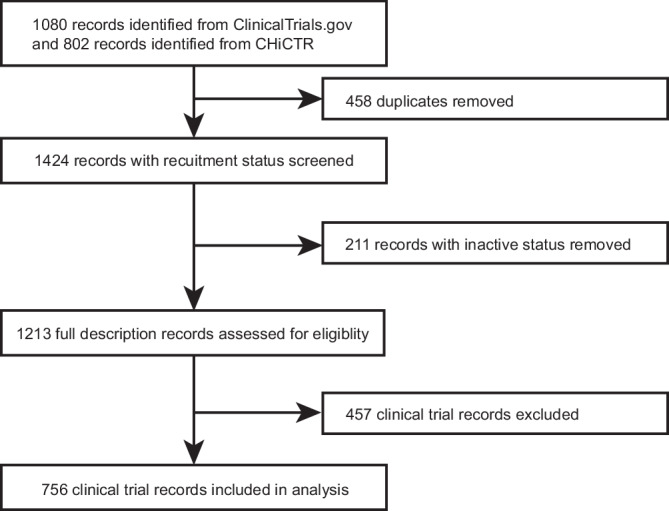
Trial identification. Flowchart of the selection process of registered clinical trials.

Our systematic search of ChiCTR and Clinicaltrial.gov identified a total of 756 registered DTx trials in China from 2014 to mid-2024, with 610 from ChiCTR and 146 from ClinicalTrials.gov (Fig. 1). Among these, 27 duplicate trials appearing in both registries were categorized under ChiCTR. Of these trials, 718 (95.0%) were interventional and 38 (5.0%) were observational (Table 1). 373 (49.3%) trials did not provide information on study phase. 316 (41.8%) trials were in early phase 1, representing the biggest proportion of all trials. The most common source of funding was government (277, 36.6%), followed by hospital/university (255, 33.7%), self-fund (176, 23.3%), and industry (42, 5.6%). Tertiary hospitals were involved in 594 (78.6%) trials, secondary hospitals in 42 (5.6%) trials, primary hospitals in 34 (4.5%) trials, community-based organizations in 72 (9.5%) trials, and others in 67 (8.7%) trials. 541 (71.6%) trials employed two arms. Only 77 (16.8%) studies used three or more.

**Table 1:** Main characteristics of the included trials (*N* = 756)

	Number	%
General characteristics		
Study type		
Interventional	718	95.0
Observational	38	5.0
Study phase		
Early phase 1	316	41.8
Phase 1	49	6.5
Phase 2	3	0.4
Phase 3	1	0.1
Phase 4	14	1.9
Not specified	373	49.3
Source of funding		
International agencies	6	0.8
Government	277	36.6
Hospital/university	255	33.7
Industry	42	5.6
Self-funded	176	23.3
Type of implementation site		
Tertiary hospital	594	78.6
Secondary hospital	42	5.6
Primary hospital	34	4.5
Community-based organization	72	9.5
Others	67	8.7
Application setting		
Non-hospital	658	87.04
Hospital	98	12.96
Number of arms		
1	86	11.4
2	541	71.6
≥ 3	77	16.8
Not specified	2	0.2
Intervention characteristics		
Model of care		
Adjunctive	121	16.0
Guided	296	39.2
Fully-automated	339	44.8
Purpose of intervention		
Prevention	397	52.5
Treatment	288	38.1
Management	71	9.4
Inclusivity		
Gender		
Both male and female	654	86.5
Male only	28	3.7
Female only	70	9.3
Not specified	4	0.5
Ethnic		
Han only	9	1.2
Not specified	747	98.8
Quality		
Total sample size		
≤100	314	41.5
101–200	217	28.7
201–500	109	14.5
>500	116	15.3
Duration of study (month)		
≤6	79	10.4
(6, 12]	167	22.1
(12, 24]	256	33.9
>24	244	32.3
Not specified	10	1.3
Number of Sites		
1	614	81.2
> 1	142	18.8
Randomization		
Randomized	673	89.0
Non-randomized	45	5.0
Not specified	38	5.0
Blinding		
Blinding	218	28.8
Open label	168	22.2
Not specified	370	48.9
Application of a digital placebo		
Yes	32	4.2
No	724	95.8

**Fig. 2:**
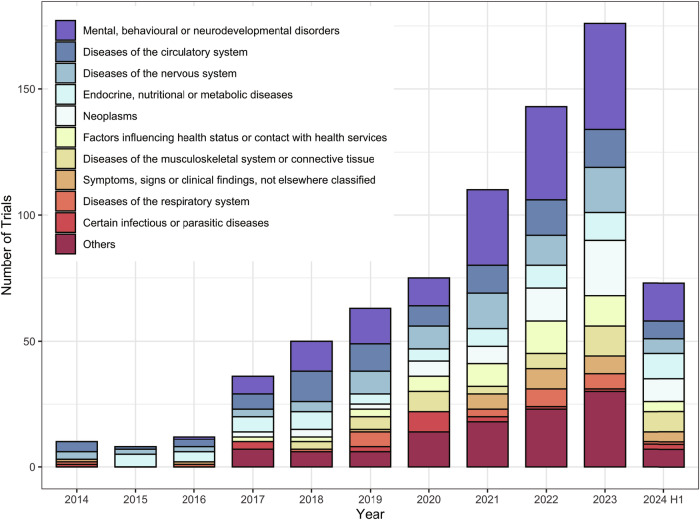
DTx trials in China by medical condition category and registration year. This figure presents the total number of DTx clinical trials in China from 2014 to the first half of 2024, categorized by medical condition category based on ICD-11 level-1 items.

Figure 2 shows a steady increase in the total number of DTx trials from 2014 to 2023. Notably, two significant surges were observed with a 200% growth rate in 2017 and a 46.7% growth rate in 2021 (Supplementary Table 2). The compound annual growth rate (CAGR) of the trials from 2018 to 2023 was 23.6%.

**Fig. 3:**
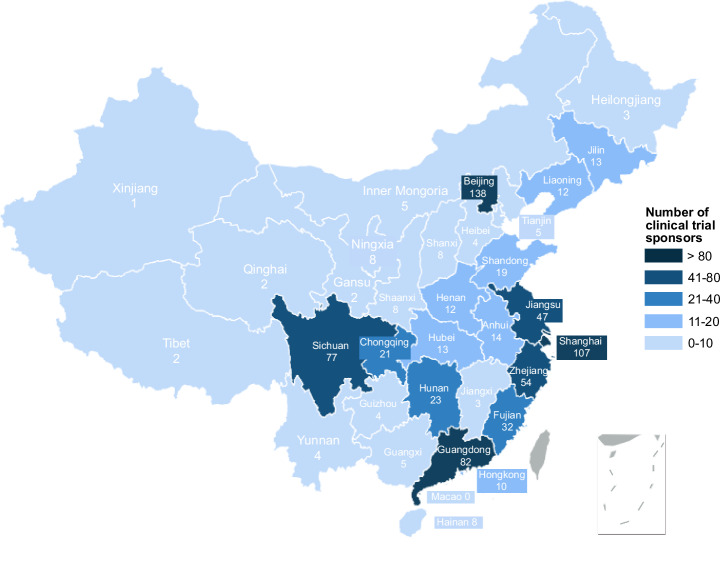
Distribution of DTx clinical trials in China. This figure shows the number of registered DTx clinical trials in China’s mainland provinces, Hong Kong Special Administrative Region, and Macau Special Administrative Region, by the location of the trial sponsor, from 2014 to the first half of 2024. Registered trials in Taiwan were not specifically included in this search.

The provinces with the most DTx clinical trial sponsors (Fig. 3) were Beijing (*n* = 138), Shanghai (*n* = 107), Guangdong (*n* = 82), Sichuan (*n* = 77), and Zhejiang (*n* = 54). Two-thirds of the trial sponsors were located in eastern China.

### Characteristics of interventions in registered trials

Table 1 shows 339 (44.8%) trials involved a fully automated DTx, 296 (39.2%) involved a DTx-guided approach, and 121 (16.0%) involved DTx as adjunctive to physician-led services. Trials involving a DTx-guided approach outnumbered those with automatic DTx before 2019, but were overtaken by the latter from 2020 onwards (Supplementary Fig. 1). Most (397, 52.5%) trials focused on disease management, followed by 288 (38.1%) on treatment and 71 (9.4%) on prevention. However, trials on treatment saw a substantial increase, surpassing trials on prevention in 2018 and peaking at 44% in 2022 (Supplementary Table 3). The number of trials on prevention has remained low in the past decade (Supplementary Fig. 1). Most adjunctive models are designed for treatment purposes, while guided and fully automated models are predominantly designed for management purposes (Supplementary Fig. 2). Meanwhile, 658 (87.0%) studies are designed for non-hospital settings (i.e. community or home), with only 98 (13.0%) hospital-based studies (Table 1).

The five most commonly targeted medical condition categories of DTx trials were “mental, behavioral, or neurodevelopmental disorders” (*n* = 169, 22.4%), “circulatory system diseases” (*n* = 92, 12.2%), and “nervous system diseases” (*n* = 82, 10.8%), “endocrine, nutritional or metabolic diseases” (*n* = 68, 9.0%) and “neoplasms” (*n* = 64, 8.5%) (Supplementary Table 4). While treatment-oriented DTx trials predominantly focused on “mental, behavioral, or neurodevelopmental disorders”, and “disease of the nervous system”, the medical condition categories addressed by the management- and prevention-oriented DTx trials were more evenly distributed amongst the conditions (Supplementary Fig. 2). The three most studied disease areas up to 2019 were “circulatory system diseases”, “mental, behavioral, or neurodevelopmental disorders”, and “endocrine, nutritional, or metabolic diseases”, replaced by “mental, behavioral, or neurodevelopmental disorders”, “nervous system diseases”, and “neoplasms” afterwards (Supplementary Table 3).

China-based DTx trials encompassed a broad spectrum of disease stages, addressing prevention, treatment, and management across a variety of conditions and models of care. For example, trials assess the effectiveness of DTx in: reducing stress and preventing perinatal anxiety and depression through DTx-guided mindfulness exercises (ChiCTR2200061990), promoting healthy sleep habits with DTx-enabled, fully-automated training programs designed to prevent the progression of acute insomnia to chronic insomnia (ChiCTR2200066448), providing non-pharmacological pain relief for children in the PICU through provider-led DTx-supported virtual reality pain interventions (ChiCTR2300073767), improving social and communication skills in children with autism through DTx-enabled, fully-automated behavioral therapy (NCT0590155), as well as improving muscle strength through DTx-guided remote rehabilitation for patients with sarcopenia (ChiCTR2300077207).

### Characteristics of participants

654 (86.5%) enrolled both male and female participants, as compared to 70 (9.3%) female-only trials, 28 (3.7%) male-only trials, and 4 trials leaving gender unspecified (Table 1). Age was not reported in 98 (13%) trials. 579 (76.6%) trials excluded children (>18 years) and 77 (10.2%) of these trials excluded the elderly (<60 years). Of the trials that did not exclude children or the elderly, 470 (62.2%) specified an upper age limit with a mean age of 75.3 (SD: 11.8) years (Supplementary Table 5). Only 9 (1.2%) trials specified the ethnicity of participants, all of which included only Han ethnicity.

### Methodological issues of registered trials

The median sample size of the trials was 120 (IQR: 80–264) (Supplementary Table 6). However, the median sample size fell from 156 in 2018 to 113 in 2023. While the number of larger trials (with sample sizes over 200) has grown slowly, the much faster increase in smaller trials (with sample sizes below 200) has driven the overall decrease in median sample sizes (Supplementary Table 7). On average, the median duration was 18.4 months among trials that reported duration. 500 (66.1%) trials applied a study duration over 1 year, with 79 (10.5%) anticipated to finish within six months. The median duration decreased from 21.8 months in 2018 to 15.3 months in 2023. There were 614 (81.2%) single-center trials and 142 (18.8%) multi-center ones. The mean number of sites is 1.6, which dropped from 2.0 in 2018 to 1.5 in 2023. 89.0% of all trials reported randomization, with the proportion of randomized trials remaining stable over the years. Only 218 (28.8%) trials reported blinding, though the proportion improved significantly from 10.0% in 2018 to 44.9% in 2023. 724 (95.8%) trials did not use a digital placebo—usually an interface such as virtual reality and app, etc., similar to that of the studied DTx, but with different content.

Table 2 summarizes the risk of bias (RoB) of the 610 ChiCTR-registered studies (532 randomized and 78 non-randomized). Among these, 110 (18%) were at low overall RoB, 58 (9%) high overall RoB, and 442 (72%) unclear. 47% exhibited studies assessed are at unclear (43%) or high (4%) risk of selection bias. 55% of studies were at unclear (51%) or high (4%) RoB due to deviations from intended interventions. RoB in outcome measurement were unclear (37%) or high (2%) for 39% of the studies. RoB varied across the five most commonly studied medical condition categories, with “circulatory system diseases” and “endocrine, nutritional, or metabolic diseases” more likely than other groups to have low RoB.

**Table 2:** Risks and sources of bias of included trials

Risk level	Source of bias			Overall risk of bias
	Selection bias	Bias due to deviations from intended interventions	Bias in measurement of the outcome	
Total (*n* = 610)				
Low	324 (53%)	274 (45%)	367 (60%)	110 (18%)
Unclear	262 (43%)	310 (51%)	228 (37%)	442 (72%)
High	24 (4%)	26 (4%)	15 (2%)	58 (9%)
Mental, behavioral, or neurodevelopmental disorders (*n* = 125)				
Low	60 (48%)	49 (39%)	74 (59%)	18 (14%)
Unclear	63 (50%)	72 (57%)	50 (40%)	102 (81%)
High	2 (2%)	4 (3%)	1 (1%)	5 (4%)
Circulatory system diseases (*n* = 74)				
Low	23 (64%)	40 (54%)	49 (66%)	18 (24%)
Unclear	47 (31%)	28 (38%)	25 (34%)	46 (62%)
High	4 (5%)	6 (8%)	0 (0%)	10 (14%)
Nervous system diseases (*n* = 72)				
Low	28 (39%)	29 (40%)	55 (76%)	10 (14%)
Unclear	43 (60%)	39 (54%)	16 (22%)	58 (81%)
High	1 (1%)	4 (6%)	1 (1%)	4 (6%)
Endocrine, nutritional, or metabolic diseases (*n* = 55)				
Low	28 (51%)	30 (55%)	37 (67%)	16 (29%)
Unclear	26 (47%)	25 (45%)	18 (33%)	38 (68%)
High	1 (2%)	0 (0%)	0 (0%)	1 (2%)
Neoplasms (*n* = 52)				
Low	31 (40%)	24 (46%)	21 (40%)	7 (13%)
Unclear	21 (60%)	25 (48%)	23 (44%)	35 (67%)
High	0 (0%)	3 (6%)	8 (15%)	10 (19%)

Selection bias: refers to bias arising from how participants are allocated into study groups. In randomized studies, it can result from flaws in the randomization process, while in non-randomized studies, it may stem from the selective inclusion or exclusion of participants based on certain characteristics. Bias due to deviations from intended interventions: refers to bias arises when there are discrepancies between the interventions participants actually receive and those intended by the study design, potentially distorting the assessment of the intervention’s effect. Bias in the measurement of outcomes: refers to bias arising from inaccuracies or inconsistencies in how outcomes are assessed, which can introduce systematic errors and affect the validity of the findings.

## Discussion

Previous studies about DTx trials neglected a key registry where most Chinese clinical trials are registered. We conducted a study of the DTx trial landscape in China to complement these findings about DTx trials globally. We observed a significant increase in the number of DTx trials over the past decade, consistent with the global trend, but at a faster growth rate than observed internationally^19,20,22^. However, over 70% of China-based trials received funding from government agencies, hospitals, and universities, suggesting that industrial investors are cautious about DTx.

Previous studies have given limited attention to intervention details such as models of care and specific intervention purposes. We identified a shift in the dominant focus of DTx trials from a primarily DTx-guided model of care to a fully automated model in China in recent years. While we were unable to isolate the effects of artificial intelligence (AI) applications in such a transition, there were clear signs of the important role of AI in recent trials. For example, DTx trials used AI to prompt patients about potentially inadequate preparation for colonoscopy and to guide further actions (ChiCTR2300071917), to improve effectiveness, quality, and safety of diabetic management in community (NCT06118671, ChiCTR2300068952), and to accelerate remote rehabilitation for patients recovering from isolated vertical meniscus tears (ChiCTR2300070582). Such a transition suggests a prospect that healthcare facilitated by DTx may be less dependent on professional healthcare providers in the future. Meanwhile, more than half of DTx trials overall involved a combined DTx-professional service (either DTx-guided or professional-led), emphasizing the importance of preparing healthcare professionals for working with DTx in a human-machine symbiosis.

Six out of every seven DTx trials are designed for community and home care settings, yet most were conducted by tertiary hospital researchers in eastern China. Such a hospital-centric model of DTx trial reflects the lack of primary care research capacity in China and the concentration of clinical research capacity in the eastern China. It potentially means neglect of the perspective of primary care and eventually restricts the ability of DTx to strengthen primary care, which would be the more suitable level of care to provide community-oriented, people-centered care. We also found a predominant focus on treatment and management, with consistently low proportions of trials on prevention, which contradicts the Healthy China policy’s emphasis on early disease prevention^8^.

Consistent with previous assessment of global DTx trials^22^, our study shows a high concentration of DTx trials related to mental, behavioral, or neurodevelopmental disorders. While circulation system diseases and cancers are the leading causes of disease burden in China^23^, they represent a relatively minor share within DTx trials. It appears that DTx may be more of a complementary role in healthcare delivery, rather than an alternative to current care model. As the care for the top causes of disease burden tends to be the ones better financed^24–26^, DTx and their trials may face restrictions in fundraising.

Previous research has shown some ethnic disparities in healthcare utilization and outcome, e.g. in maternal and child health, dental care, and diabetes and prediabetes^27–30^, indicating value in specific inclusion of ethnic minorities in clinical trials. Although DTx trials in China did not explicitly exclude ethnic minority groups, the lack of intentional inclusion may represent a missed opportunity to address their unique needs within these trials. The high prevalence of age-based exclusion criteria raises concerns related to ageism in clinical trials and casts doubts of applicability to a wider population.

The median sample size of Chinese DTx trials stayed above the global average, though it decreased over the past five years in contrast to the global rising trend^22^. The reduction in median sample size of the Chinese trials reflects the recent surge in the number of trials, many of which are at early stages with smaller sample sizes. The fast growth of number of trials with samples below 200 has contributed to a decline in median sample sizes. This trend may reflect the exploratory nature of recent DTx studies and/or smaller funding allocation per trial. Additionally, both trial durations and number of sites have decreased, with median durations in China being longer, and the median number of collaborators lower than global counterparts^21^. The proportion of randomized studies in China is higher, but the proportion of blinded studies is lower compared to global averages^22^. We observed a low (4%) use of digital placebos in DTx trials in China, mirroring the global situation^19^. This may be explained by the inherent challenges of implementing blinding and digital placebos effectively^31^. In general, the design quality of trials in China appears to be not inferior if not better than that of international ones, where only a small proportion of DTx studies globally is comprehensively well-designed^19^.

To the best of our knowledge, RoB assessment has not been conducted across DTx trials for all health conditions. Recognizing that reliance on published papers prevents timely assessment of the RoB of ongoing trials, we leveraged available registry data on ChiCTR for RoB assessment. Our findings suggest that most trials in China at unclear or high RoB in their design, consistent with the finding of a previous systematic review on digital mental health interventions by Lattie and colleagues, which analyzed 89 published studies and identified a moderate-to-severe risk of bias in many trials^32^.

Considering what we have found in the Chinese trials, there might be several implications for future practice and research. First, there is room for further improvement of trial designs to reduce biases, particularly in improving the use of blinding and digital placebo. Coordinated efforts to enhance DTx-specific research capacity are needed, such as formulating methodological guidelines for DTx studies and creating research training programs. Second, the capacity of primary care researchers should be strengthened to enable their leadership in DTx trials to reflect the perspective of community-oriented and primary care. Third, future trials in China should aim at more explicit inclusion of the western part of the country and ethnic minorities. Fourth, more DTx trials on preventive care and medical conditions that cause high disease burden should be encouraged. Fifth, as many DTx interventions still require professional support in service provision, health workers should be prepared to work in a human-machine symbiosis environment in the future.

Our studies have several limitations. First, we did not include data from trial registries other than the ChiCTR and Clinicaltrial.gov, which means a potential underestimation of China-based trials. Second, our assessment of RoB was not able to cover all China-based trials, due to the lack of relevant information on ClinicalTrials.gov regarding randomization and blinding procedure. Third, we were not able to compare the quality of trials registered on ChiCTR and ClinicalTrials.gov due to the limited scope of this study, while comparing trial characteristics across registries might offer valuable insights into inter-registry differences in trial quality. Last but not least, because we only considered the trial registration data but did not examine published protocols or papers, the assessment of the quality of study designs may not reflect the actual quality of trials as implemented.

## Methods

### Search strategy and selection criteria

We performed a search of DTx trials registered through the ClinicalTrials.gov (English) and Chinese Clinical Trials Registry (ChiCTR) (Chinese) from January 1, 2014, to June 30, 2024. We used the Chinese versions of the terms “digital therapeutics”, “telehealth”, “internet”, “mobile health”, “virtual reality”, “application”, and “mini program” to identify potential DTx trials on ChiCTR, and their English equivalents for the trials based in China on ClinicalTrials.gov (see detailed search terms in Supplementary Table 8).

After removing duplicate trials within and between each of the two registries, trials with active recruitment status or completed trials were included, i.e. those not “terminated”, “withdrawn”, “suspended”, “unknown”, or “no longer available”. After that, we assessed the full description of trials and excluded the ones, according to the DTx Value Assessment & Integration Guide (DTA, 2022)^33^, where: (1) the main purpose of intervention could not be categorized as disease prevention, treatment or management (i.e. general health promotion, wellness, fitness, or solely informing, monitoring, diagnosing, or offering insights to clinicians); (2) DTx was not delivered as part of the intervention to the patient (i.e. solely by a clinician or a medication independent of the software). Duplicated trials appearing in both registries were assigned to the ChiCTR category.

### Variables, data extraction, and coding

Considering the information available on both registries, we assessed the priorities, quality, and inclusivity of trials using a set of indicators.

First, we evaluated a set of basic trial characteristics using the information provided on the registries: registration date, study type, study phase, source of funding, type of implementation site, number of arms, as well as the location of trial sponsors.

Second, we categorized the trials based on their textual description of intervention by: (1) model of care—“adjunctive” (i.e. provider-led DTx-supported), “guided” (i.e. DTx-led provider-supported) or “fully automated” (i.e. independent of provider involvement), according to a commonly used typology for behavioral intervention technology^3^; (2) purpose of intervention—“prevention”, “treatment” or “management”; and (3) application setting—“hospital” or “non-hospital”; (4) targeted medical condition category according to ICD-11 (see Supplementary Table 4).

Third, we assessed the quality of study design by reviewing the recorded sample size, duration of study, number of sites, randomization, blinding, and application of a digital placebo, as well as RoB. We assessed the RoB based on information available on the ChiCTR, namely, study design, randomization procedure, blinding procedure, inclusion criteria and exclusion criteria for participants, intervention and comparison, and primary outcomes (outcome, type of outcome, measurement time point of outcome, and measurement). Due to the lack of relevant information on ClinicalTrials.gov regarding randomization and blinding procedure, necessary for an RoB assessment, only studies registered on ChiCTR were included for our RoB assessment. In other words, we did not attempt to compare the RoB for trials registered in ChiCTR relative to those registered in ClinicalTrials.gov.

RoB was assessed using criteria from the Cochrane Handbook for Systematic Reviews of Interventions (version 6.4, 2023), specifically the RoB assessment for randomized trials and non-randomized studies^34,35^. The potential RoB was assessed in three key domains for both randomized and non-randomized studies: (1) selection bias (“bias arising from the randomization process” for randomized studies and “bias in the selection of participants into the study” for non-randomized studies); (2) bias due to deviations from intended interventions; (3) bias in the measurement of outcomes. RoB in each domain was rated as high, low, or unclear, based on trial descriptions. As our focus was trial design rather than result, we excluded the domains of biases “due to missing outcome data” and “in selection of the reported result” for all studies. For non-randomized studies, we further excluded biases “due to confounding” and “in classification of interventions”. Trials judged as low RoB in all domains were classified as low overall RoB; trials with any high risk were classified as high overall risk; and trials with any unclear risk were classified as unclear overall risk.

Fourth, we assessed the inclusivity of trials by examining the recorded gender, age, and ethnic composition of participants.

For variables other than RoB, two authors (X.X. and Z.W.) independently conducted data extraction and coding. The results of this initial extraction and coding were discussed between the two reviewers, with remaining disagreements resolved by discussion amongst the research team. Two authors (X.X. and W.L.) assessed RoB independently for each trial. Disagreements were resolved through discussion or by involving a third author (Z.W.).

### Data acquisition, analysis, and visualization

We used Python 3.8.13 (Python Software Foundation, Delaware, USA) with the pandas package version 1.3.5 and seaborn package version 0.12.0 to download, code, and perform descriptive analyses of the data^36,37^. All codes are accessible in the following GitHub repository [https://github.com/kirihsia/An-assessment-of-digital-therapeutics-trials-in-China.git]. A Sankey diagram was used to visualize the changing characteristics of interventions and temporal trend of number of registered trials, model of care, purpose of intervention and disease category. Maps were drawn to describe the provincial distribution of trial sponsors within China.

## Supplementary information

Supporting information

## Data Availability

The datasets generately and analysed during the current study are available in the An-assessment-of-digital-therapeutics-trials-in-China repository, https://github.com/kirihsia/An-assessment-of-digital-therapeutics-trials-in-China.

## Code availability

The underlying code for this study is available in An-assessment-of-digital-therapeutics-trials-in-China repository and can be accessed via this link https://github.com/kirihsia/An-assessment-of-digital-therapeutics-trials-in-China.
