# Supplementary material for: Assessment of priorities, quality, and inclusivity of digital therapeutics trials in China
Source: NPJ Digit Med. 2025 Feb 4;8:83. doi: 10.1038/s41746-025-01477-6 (PMC11799303; doi:10.1038/s41746-025-01477-6)
Supplement: Supplementary file 1 — Supporting information [file 41746_2025_1477_MOESM1_ESM.pdf]

Supplementary Information

Supplementary Table 1: Characteristics of previous global assessments on DTx trials

ID	Title	Authors, year of publication	Definition of DTx adopted by the article	Objects of study	Search strategy	Eligibility criteria	Number of clinical trials included
1	Studies Of Prescription Digital Therapeutics Often Lack Rigor And Inclusivity	Kumar et al, 2024	*Digital therapeutics aim to treat, manage and prevent diseases and clinical disorders. Prescription digital therapeutics are a growing subset of digital therapeutics; they are regulated by the FDA under the SAMD framework and require prescriptions from clinicians.*	Study results from multiple sources, including ClinicalTrials.gov, PubMed, FDA, DTA, and manufacturers' websites	Use product names as search terms. The authors identified the brand names of prescription DTx from third-party sources and located their product codes in FDA databases. Using these codes to discover additional DTx products, the authors thereafter found all related clinical studies.	Inclusion criteria: Only FDA-authorized prescription DTx are considered for inclusion. Both premarket and postmarket studies of FDA-authorized prescription DTx are included.<br><br>No explicit exclusion criteria were reported.	68
2	A comprehensive survey of the clinical trial Landscape on digital therapeutics	Yao et al, 2024	*A digital therapeutic is an emerging and groundbreaking medical intervention that utilizes health software to treat or alleviate various diseases, disorders, conditions, or injuries.*	Trial records on ClinicalTrials.gov	Using the following terms: (1) Intervention/Treatment: "digital therapeutics," "digital intervention," "digital medicine," or "DTx"; (2) Other terms: "digital therapeutics" or "DTx.", to identify related trials registered on ClinicalTrials.gov by August 8, 2023. No specific launch or end date.	Inclusion criteria: (i) completed; (ii) delivered through software; (iii) delivered via a technology platform, medical device, or medication; and (iv) aimed at treating, managing, or preventing a disease or condition.<br><br>Exclusion criteria: (i) duplicates from both search strategies; (ii) incomplete (e.g., not recruiting, suspended, terminated); (iii) not using software as an intervention; and (iv) conducted solely by clinicians providing only notification, monitoring, or diagnostic information.	280

3	Characterisation of digital therapeutic clinical trials: a systematic review with natural language processing	Miao et al, 2024	*Digital therapeutics are a somewhat novel class of US Food and Drug Administration-regulated software that helps patients prevent, manage, or treat disease.*	Trial records on ClinicaTtrials.gov	Use of a set of 27 search terms related to DTx, including “digital therapeutic”, “digital therapy”, “smartphone”, “mobile app”, and “video game”, to identify studies with start dates after 2010 or expected completion dates after 2030, registered on ClinicalTrials.gov as of Aug 3, 2022.	Inclusion Criteria: Only trials registered for FDA-regulated devices were included.<br><br>Exclusion Criteria: Basic science studies, as well as trials that were terminated, withdrawn, suspended, or had unknown status, were excluded to focus on active trials.	449
4	Tracing Digital Therapeutics Research Across Medical Specialties: Evidence from ClinicalTrials.gov.	Masanneck and Stern, 2024	*Digital therapeutics, evidence-based software interventions for preventing, managing, or treating medical disorders.*	Trial records on ClinicalTrials.gov	Use the following keyword “digital therapy OR digital therapies OR digital intervention OR digital interventions OR digital therapeutic OR digital therapeutics OR DTx” to identify related trials registered on ClinicalTrials.gov by May 22, 2023.	Inclusion Criteria: Trials with recruitment statuses of “Recruiting,” “Active, not recruiting,” “Completed,” or “Enrolling by invitation” launched between 2005 and 2022 (inclusive) were included.<br><br>Exclusion Criteria: Trials that were withdrawn, suspended, terminated, or not yet recruiting were excluded.	5,889

Note: EMA, European Medicines Agency; FDA, Food and Drug Administration; SAMD, Software as a Medical Device. Premarket studies refer to those initiated before FDA authorization of a medical device, regardless of its pathway. Postmarket studies refer to the studies initiated after FDA authorization.

Supplementary Table 2: Annual count of new DTx clinical trials and compound annual growth rate (CAGR) from 2018 to 2023.

Registry year	2014	2015	2016	2017	2018	2019	2020	2021	2022	2023	2024 H1
Number	10	8	12	36	50	63	75	110	143	176	73
CAGR (2018-2023)	23.6%										

Supplementary Figure 1: Numbers of DTx clinical trials from ChiCTR and ClinicalTrials.gov (2014-the first half of 2024). a Numbers of DTx clinical trials by model of care. **b** Numbers of DTx clinical trials by purpose of intervention.

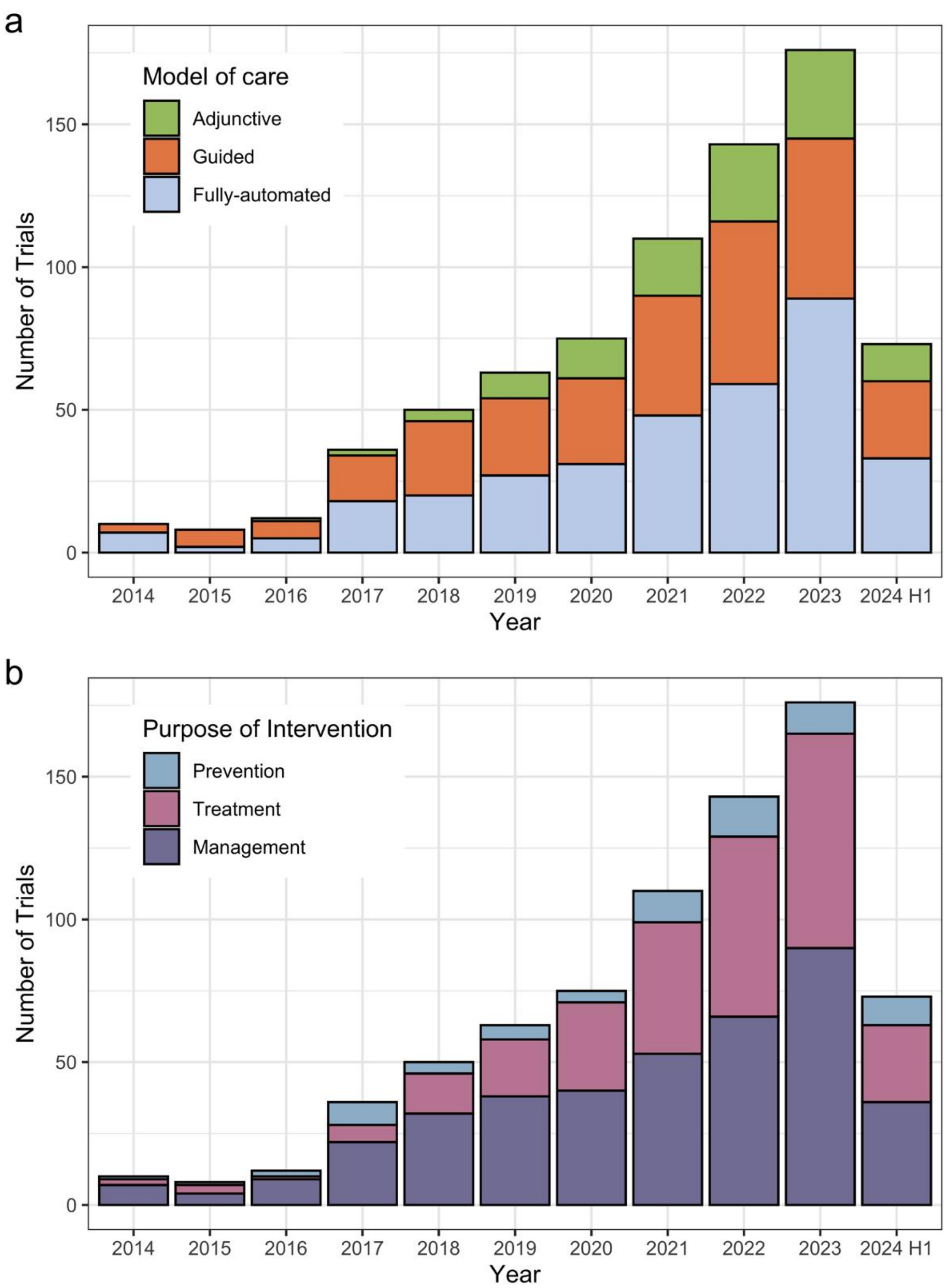

Supplementary Figure 2: Trends in Digital Therapeutics (DTx) Clinical Trials by Registry, Year, Model of Care, Purpose of Intervention, and Medical Condition Category. The Sankey diagram visualizes trends in DTx trials across five **dimensions**: **registry** (ChiCTR, Clinicaltrial.gov)**, year of registration, model of care** (adjunctive, DTx-guided, fully automated), **Purpose of intervention** (prevention, treatment, management), and **medical condition category** (displaying only categories with more than 10 trials). Each **node** within a dimension represents a category (e.g., a specific year, model of care type, or intervention purpose) and shows the distribution of trials within that category. **Lines** between nodes represent transitions of trial characteristics across dimensions, with line thickness indicating the relative number of trials moving from one category to the next. The flow from left to right displays how trials over the years have distributed across models of care, purposes of intervention, and, finally, specific medical condition categories. Notable trends include the increasing proportion of fully automated care models over time, the tendency for adjunctive care models to align with treatment purposes, while guided and fully automated models predominantly align with management purposes. The diagram also highlights the prominence of trials focused on mental, behavioural, and neurodevelopmental disorders, with a significant flow from treatment purposes into this medical condition category. Data are based on a total of 756 DTx trials identified from ChiCTR and ClinicalTrials.gov between 2014 and mid-2024.

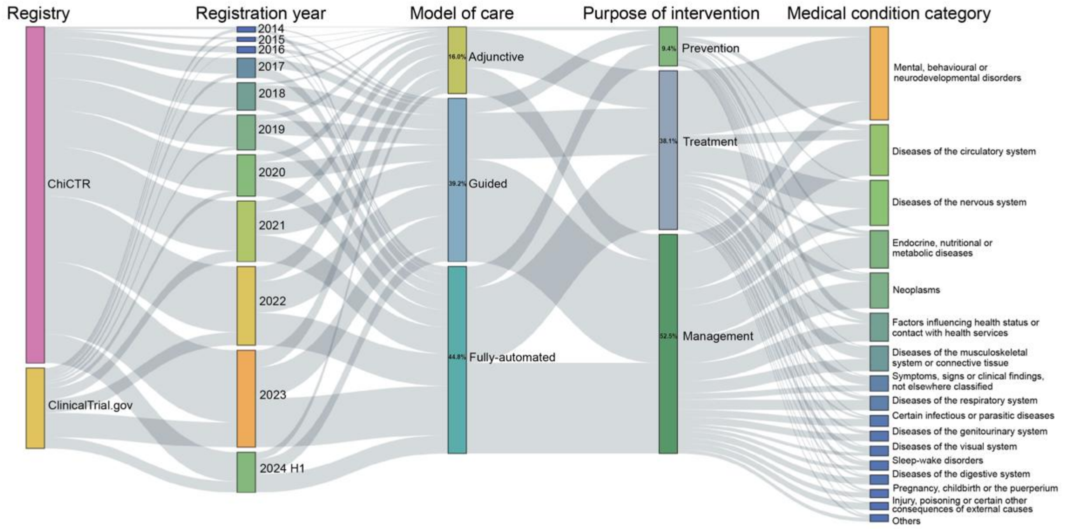

Supplementary Table 3 :Annual distribution of DTx clinical trials over the past decade by model of care, purpose of intervention, and ICD-11 medical condition category.

Characteristic	Year of registration										
	2014	2015	2016	2017	2018	2019	2020	2021	2022	2023	2024 H1
Model of care											
Adjunctive	0	0	1	2	4	9	14	20	27	31	13
Fully automated	7	2	5	18	20	27	31	48	59	89	33
Guided	3	6	6	16	26	27	30	42	57	56	27
Purpose of intervention											
Prevention	1	1	2	8	4	5	4	11	14	11	10
Treatment	2	3	1	6	14	20	31	46	63	75	27
Management	7	4	9	22	32	38	40	53	66	90	36
ICD-11 medical condition category											
Mental, behavioural or neurodevelopmental disorders	0	0	1	7	12	14	11	30	37	42	15
Diseases of the circulatory system	4	1	3	6	12	11	8	11	14	15	7
Diseases of the nervous system	3	2	2	3	4	9	9	14	12	18	6
Endocrine, nutritional or metabolic diseases	0	5	4	6	7	4	5	7	9	11	10
Neoplasms	0	0	0	2	3	2	6	7	13	22	9
Factors influencing health status or contact with health services	0	0	0	2	2	3	6	9	13	12	4
Diseases of the musculoskeletal system or connective tissue	0	0	0	0	3	5	8	3	6	12	8
Symptoms, signs or clinical findings, not elsewhere classified	1	0	1	0	0	1	0	6	8	7	4
Diseases of the respiratory system	1	0	1	0	1	6	0	3	7	6	1
Certain infectious or parasitic diseases	1	0	0	3	0	2	8	2	1	1	2
Diseases of the visual system	0	0	0	1	1	1	2	2	3	5	3
Diseases of the genitourinary system	0	0	0	0	3	2	3	1	5	4	0
Sleep-wake disorders	0	0	0	1	0	2	1	3	5	5	0
Diseases of the digestive system	0	0	0	0	0	1	2	4	4	3	3
Pregnancy, childbirth or the puerperium	0	0	0	3	0	0	1	3	3	4	1

Injury, poisoning or certain other consequences of external causes	0	0	0	1	2	0	2	1	1	6	0
Others	0	0	0	1	0	0	3	4	2	3	0

Note: The medical condition categories of clinical trials with more than 10 trials are shown separately.

Supplementary Table 4: Distribution of DTx clinical trials by ICD-11 medical condition category with examples.

Medical condition category	Number (%)	Example
Mental, behavioural or neurodevelopmental disorders	169 (22.4%)	"Mild cognitive impairment", "Depressive disorder", "Schizophrenia", "Autism spectrum disorder"
Diseases of the circulatory system	92 (12.2%)	"Hypertensive diseases", "Heart failure", "Venous thromboembolism"
Diseases of the nervous system	82 (10.8%)	"Stroke", "Parkinson disease"
Endocrine, nutritional or metabolic diseases	68 (9.0%)	"Diabetes", "Polycystic ovary syndrome"
Neoplasms	64 (8.5%)	"Breast cancer", "Lung cancer"
Factors influencing health status or contact with health services	51 (6.7%)	"Care involving peritoneal dialysis", "Physical rehabilitation"
Diseases of the musculoskeletal system or connective tissue	45 (6.0%)	"Osteoarthritis of knee", "Sarcopenia"
Symptoms, signs or clinical findings, not elsewhere classified	28 (3.7%)	"Pain", "Tinnitus"
Diseases of the respiratory system	26 (3.4%)	"Chronic obstructive pulmonary disease"
Certain infectious or parasitic diseases	20 (2.6%)	"HIV", "COVID-19"
Diseases of the visual system	18 (2.4%)	"Amblyopia", "Glaucoma"
Diseases of the genitourinary system	18 (2.4%)	"Chronic kidney disease", "Neurogenic bladder"
Sleep-wake disorders	17 (2.2%)	"Chronic insomnia", "Obstructive sleep apnoea"
Diseases of the digestive system	17 (2.2%)	"Gastric ulcer", "Non-alcoholic fatty liver disease"
Pregnancy, childbirth or the puerperium	15 (2.0%)	"Diabetes mellitus in pregnancy", "Preterm labour"
Injury, poisoning or certain other consequences of external causes	13 (1.7%)	"Rotator cuff injury", "Injury of spinal cord"
Diseases of the skin	7 (0.9%)	"Acne", "Atopic dermatitis"
External causes of morbidity or mortality	1 (0.1%)	"Fall or jump with undetermined intent"
Diseases of the immune system	1 (0.1%)	"Lupus erythematosus"
Diseases of the ear or mastoid process	1 (0.1%)	"Deaf"
Developmental anomalies	1 (0.1%)	"Congenital heart disease"
Conditions related to sexual health	1 (0.1%)	"Male early ejaculation"
Certain conditions originating in the perinatal period	1 (0.1%)	"Neonatal Jaundice"

Supplementary Table 5: Age characteristics of participants of the included trials (N=756)

	**Number**	**%**
Trials without age limit	10	1.3
Trials only including children (> 18)	69	9.1
Trials excluding children (> 18)	579	76.6
Trials excluding elderly (< 60)	77	10.2
Trials including elderly (< 60)	502	66.4
Trials with an upper age limit	470	62.2
Trials without an upper age limit	32	4.2
Not specified	98	13.0

Supplementary Table 6: Key design parameters of DTx clinical trials over the past decade.

Year of registration	Mean number of sites (SD)	Randomized (%)	Blinding (%)	Median duration (IQR)	Median sample size (IQR)
Total	1.6 (1.7)	673 (89.0%)	218 (28.8%)	18.4 (11.9-30.4)	120 (80-264)
2014	1.6 (1.3)	10 (100%)	3 (30%)	27.9 (17.0-32.7)	280 (122.5-1000)
2015	1.8 (1.4)	7 (87.5%)	1 (12.5%)	25.9 (21.4-29.4)	191 (97-308)
2016	1.8 (2.3)	11 (91.7%)	1 (8.3%)	21.3 (10.1-37.5)	175 (45.75-404.5)
2017	1.8 (2.2)	32 (88.9%)	4 (11.1%)	23.3 (15.6-31.5)	256 (120-1000)
2018	2.0 (2.0)	42 (94.0%)	5 (10.0%)	21.8 (12.2-36.5)	156 (85-270)
2019	1.7 (1.7)	54 (85.7%)	12 (19.0%)	24.3 (12.7-36.5)	148 (80-300)
2020	2.0 (2.3)	63 (85.1%)	14 (18.7%)	24.3 (12.2-33.1)	164 (73-420)
2021	1.6 (1.6)	102 (92.7%)	20 (18.2%)	17.0 (12.2-27.3)	115 (80-200)
2022	1.5 (1.6)	134 (93.7%)	35 (24.5%)	18.3 (10.6-28.5)	112 (80-200)
2023	1.5 (1.4)	153 (86.9%)	79 (44.9%)	15.3 (9.1-24.4)	113 (78-224.5)
2024 H1	1.5 (1.7)	65 (89.0%)	44 (60.3%)	15.2 (9.2-29.9)	100 (64-200)

Note: The proportion of randomized and blinded clinical trials in each year was calculated using the annual total as the denominator.

Supplementary Table 7: Classification of DTx Clinical Trials by Sample Size Over the Past Decade.

Year of registration	Sample size			
	**≤ 100**	**101-200**	**201-500**	**> 500**
Total	314	217	109	116
2014	2	3	1	4
2015	3	3	0	2
2016	4	3	3	2
2017	7	10	6	13
2018	18	17	8	7
2019	23	16	13	11
2020	27	18	13	17
2021	47	37	19	7
2022	68	41	18	16
2023	76	52	23	25
2024 H1	39	17	5	12

Supplementary Table 8: DTx-related search terms from ChiCTR and ClinicalTrials.gov.

Registry (language)	Search terms
ChiCTR (Chinese)	“远程”, “互联网”, “移动”, “虚拟现实”, “应用程序”, “小程序” , “数字疗法”
ClinicalTrials.gov (English)	“Telehealth”, “Long distance”, “Remote”, “Internet”, “Mobile health”, “Mobile platform”, “Virtual reality”, “App”, “Mobile app”, “Software intervention”, “Software treatment”, “Mini program”, “Mobile program”, “Digital therapeutics”, “Digital therapy”, “Digital intervention”, “Digital treatment”, “Digital health”
